# Supplementary material for: Are carbon emissions trading and green financial instruments synergistic? -Comprehensive quantitative research based on content analysis
Source: PLoS One. 2024 Mar 7;19(3):e0298601. doi: 10.1371/journal.pone.0298601 (PMC10919601; doi:10.1371/journal.pone.0298601)
Supplement: S1 Data — (ZIP) [file pone.0298601.s001.zip › Data/地级市Z_F工作报告.zip!/╕ú╜¿/╚¬╓▌╩╨/└┤╫╘╣1⁄2╓┌║┼í╛┬φ┐╦╩2╛▌═°í┐_2017.docx]

各位代表：

　　现在，我代表泉州市人民政府向大会报告政府工作，请予审议，并请政协委员和其他列席人员提出意见。

　　一、过去五年及2016年政府工作回顾

　　五年来，在中共泉州市委领导下，本届政府认真贯彻习近平总书记系列重要讲话精神和对福建工作的重要指示，全面落实中央、省和市委重要决策部署，适应把握引领经济发展新常态，深入实施市委“三大战略”，全力推进“五个泉州”建设，有为应对严峻挑战，经受住了重大考验，经济社会发展迈上新台阶。

　　过去五年，泉州经济在科学发展中跨越前行。2016年全市实现生产总值6646.6亿元、一般公共预算收入424亿元、固定资产投资3748亿元、外贸进出口1652亿元，分别比2011年增长62%、75%、145%和48%；经济总量连续18年居全省首位，人均地区生产总值达7.8万元；三次产业比重由3.6∶61.5∶34.9调整为3∶58.7∶38.3。形成纺织服装、鞋业、石油化工、机械装备、建材家居五大千亿产业集群，拥有上市公司101家、中国驰名商标152枚。

　　过去五年，泉州改革在攻坚克难中砥砺奋进。组织实施“金改”“综改”等44项国家级改革试点，农业农村、科教文卫、生态文明、社会治理、政府职能转变等重点领域改革多点突破，初步形成具有泉州特色的改革体系。获批创建国家自主创新示范区。21世纪海上丝绸之路先行区建设列入国家战略。

　　过去五年，泉州城乡在统筹融合中绽放新颜。城市环湾、向湾、同城化发展步伐加快，环湾建成区面积达214平方公里，全市城镇化率达64.5%，比2011年提高5.2个百分点。晋江新型城镇化得到习近平总书记批示肯定。美丽乡村建设全面铺开，永春被国家列入标准样板。城乡基础设施更加完善，环城高速闭合成环，实现县县通高速，迈入亿吨大港、国际机场行列。

　　过去五年，泉州文化在传承弘扬中彰显独特魅力。当选中国首个“东亚文化之都”，成功举办第十四届亚洲艺术节、“海丝”国际研讨会、中阿城市论坛，“海丝”国际艺术节永久落户泉州，成为国务院确定的“海上丝绸之路·中国史迹”申报世界文化遗产牵头城市。新增国家级文保单位11个、非遗项目6个，新增世界非遗名录1项、记忆名录1项，成为全国唯一拥有联合国教科文组织全部三大类别非遗名录的城市。

　　过去五年，泉州人民在共建共享中有了更多获得感。五年累计统筹8.6亿元资金，帮助12.6万人脱贫，城乡居民人均可支配收入分别年均增长8.9%、10.5%。全面实现县域义务教育发展基本均衡，新增中小学学位9万个、公办幼儿园学位7.5万个。医药卫生体制改革全面推进，新增医疗机构床位1.1万张。城乡医疗保险、养老保险加快从制度全覆盖走向人群全覆盖。荣膺全国文明城市、综治最高奖“长安杯”、国家生态市、全国绿化模范城市，保持国家级科技、卫生、环保、园林等城市荣誉。

　　2016年是本届政府最后一年，在市委领导下，我们坚持稳增长、控风险、谋长远，全市生产总值、一般公共预算收入、固定资产投资分别增长8%、9.2%和10%，“十三五”实现良好开局。

　　（一）精准施策力促经济稳定增长

　　打好政策“组合拳”。出台稳定工业、企业技改、兼并重组、降低成本等10多份专项政策，兑现企业扶持资金40.3亿元；争取国家和省级补助110.7亿元。深入一线帮扶。落实市委“帮扶企业发展促进项目落地”活动周。大力支持企业拓展市场，政府投资工程采购名优地产建材产品超30亿元，“手拉手”对接产能823亿元，开展泉州品牌“境外行” “海丝行” 39场。扩大有效投资。实施“重大项目前期工作活动年”，组织项目建设“百日大会战”，753个“五个一批”项目完成投资942亿元，25个补短板工程包完成投资185亿元。争取国家专项建设基金95.9亿元。全力防控金融风险。实施党政主官“一把手”工程，充分发挥金融风险预警监测和防控化解一体化平台作用，加大不良贷款处置力度，严厉打击恶意逃废债，不良贷款率控制在较低水平。

　　（二）按“路线图”推进供给侧结构性改革

　　出台落实供给侧结构性改革系列政策，组织实施重点产业转型升级路线图，全力推动产业延伸补缺、做大做强。深入实施“泉州制造2025”。“数控一代”创新应用经验在全国推广，全面完成“21211”工程，获批“中国制造2025”城市试点示范。出台国家自主创新示范区建设“1+X”政策体系，新增科技小巨人领军企业163家、发明专利1350件。实施质量品牌提升计划，新增中国驰名商标8件、中国质量奖提名奖1家，制（修）订标准51项，德化获评中国版权金奖保护奖。实施重点技改项目217个，全市完成技改投资850亿元。大力培育龙头企业。晋华集成电路、天电光电、中科植物工厂、安踏一体化产业园等重大项目开工，通达智能制造、晋江高效太阳能电池等32个制造业重点项目投产。深化产业链招商、定向招商，累计纳入全省“政民外”项目682个，总投资7205亿元。加快建平台聚人才。引进设立石墨烯产业研究院、哈工大工研院、南洋研究院等高端平台。深化“海纳百川”高端人才集聚计划，新增院士专家工作站7家，入选省高层次人才49人。实施企业家素质提升培训计划，参与企业家1.6万人次。充分释放大众创业万众创新活力，累计建设众创空间、孵化器71家，其中国家级8家。深化“第三产业发展年”。404个项目完成投资631亿元。实施“互联网+”行动计划，新上线“泉州购”“跨境通”和油化品航运电商平台，网商虚拟产业园入驻企业超7000家，全市电商交易总额2810亿元，比增39%。新增“3A”以上物流企业14家，传化公路港、顺丰物流园区等投入使用。全市接待游客5859万人次、旅游总收入717亿元，分别增长14.3%和16.1%，永春、德化获批创建国家全域旅游示范区。发展特色现代农业。完成农林牧渔业总产值357.3亿元。实施重点设施农业项目96个，面积5464亩。新增农业龙头企业81家，农民合作社、家庭农场、专业大户等新型经营主体5000家，农业“三品一标”认证38个。深沪远洋渔业作业区获准对外开放。

　　（三）先行先试深化改革扩大开放

　　抓好国家“金改区”建设。银行主渠道作用更加有为有效，全市各项贷款余额5865.5亿元；中小微企业信用信息交换共享平台帮助授信300多亿元，“无间贷”模式在全国推广。区域资本市场更加活跃，新增境内外上市企业5家、新三板挂牌企业30家；海峡股权泉州交易中心挂牌企业超500家，全省首家金融租赁公司开业。深入实施“民综”改革。发挥财政资金的撬动效应，设立运营产业投资基金及新兴产业、集成电路、高新技术等子基金。推广PPP模式，落地项目17个、总投资258.5亿元。深化工商登记制度改革，率先启动个体工商户“两证整合”，实行企业“五证合一、一照一码”，新增市场主体12万户。出台深化国有企业改革意见，组建城建、文旅集团。完成公共资源交易平台整合。实施不动产统一登记，全面推进农村土地承包经营权确权登记颁证，晋江、石狮成为国家农村住房财产权抵押贷款试点，永春成为国家农村承包土地经营权抵押贷款试点，国有林场改革深入实施。加快“海丝”先行区建设。牵头推进“海丝”联合申遗，开展14处遗产点、1处关联点的修缮保护和环境整治，我市首部实体法《泉州市海上丝绸之路史迹保护条例》获批，首发海上丝绸之路特种邮票。与“海丝”沿线国家和地区贸易额超120亿美元，与丹麦霍尔拜克市建立友好城市关系。泉州晋江国际机场旅客吞吐量达379.3万人次。完成港口吞吐量1.26亿吨、集装箱204.5万标箱，石湖5～6#泊位工程开工。泉金航线运送旅客11.5万人次。综合保税区通过考核验收。

　　（四）“点线面”加快城市建设提升

　　抓好城市关键点、精品点。501个环湾规划建设项目完成投资514亿元，泉州科技与规划馆、东海工人文化宫、大剧院、图书馆、东海大厦及泉州植物园等加快推进。建设城市交通线、景观线。城东至北峰快速通道、综合交通枢纽等项目开工，有轨电车前期工作进展顺利；中心市区公共自行车租赁系统投入使用，建设站点280个，投用自行车9000辆，骑行超500万人次；山线示范段、滨江北路水线等加快建设，“2+2”生态景观提升基本完成。打造城市示范面、展示面。编制环湾新城空间控制和城乡一体化规划，开展重要节点城市设计，建立环湾规划审批报备制度，实现中心市区单元控规全覆盖。系统谋划推进古城保护提升，修编历史文化名城保护规划，完成三朝巷、象峰巷微改造；建成开放古城规划设计展示馆、古城会客厅，完成府文庙保护利用提升工程，培育新门街文化旅游街区、苏廷玉故居等业态示范点；开通古城语音导览系统，举办首届“海丝”古城穿越徒步活动。推进城市精细管理。启动城市管理体制改革。强化城市管理综合考评，持续开展市容市貌整治。制定完善中心市区旧厂房改造政策。拆除“两违”568.2万平方米。完成中心市区超标电动自行车综合整治。加快新型城镇化步伐。22个小城市培育试点实施项目675个，完成投资383.3亿元，新增国家和省级特色小镇6个。泉港入选国家产城融合示范区。全面放开落户限制，新办理居住证59万张，同等享受基本公共服务。

　　（五）以人为本不断增进民生福祉

　　坚持把七成以上的地方财力用于保障和改善民生。36项为民办实事项目全部完成。抓好超强台风防抗和灾后重建。受“莫兰蒂”“鲇鱼”接连袭击，我市工农业和基础设施损毁严重，直接经济损失达99.2亿元。全市党政军民合力抗灾救灾，第一时间抢通抢修，及时出台实施灾后重建“1+11”方案，落实财政救灾资金4.8亿元，农村住房、交通水利、工农业生产等恢复重建工作进展顺利，受灾群众春节前可全部搬入新居。精准推进扶贫工作。出台强化精准扶贫打赢脱贫攻坚战实施意见，完成贫困人口建档立卡，实现脱贫3.3万人。完成农村危房改造2433户，造福工程搬迁8659人。建设提升农村人居环境。持续推进“千村整治、百村示范”，建设环境整治村184个、示范宜居村42个和美丽乡村景观带10条。拆除改造石结构房屋2862.6万平方米，完成公路防护工程420公里，农网改造升级投资10.3亿元。认真落实市人大关于加快规划建设白濑水利枢纽工程议案的决议，项目前期工作基本完成；七库连通、山美生态试点项目加快推进，彭村水库、向金门供水工程（大陆段）主体完工，农村5.4万人饮水安全问题得到解决。加强就业和社会保障。新增城镇就业13万人，农村劳动力转移就业6万人。职工“五险”人数增加15万人次，城乡医疗保险、养老保险待遇稳步提高，城市社区居家养老服务站实现全覆盖，建设农村老年体育活动中心20个。加强老龄、老干部和关心下一代工作。建立健全救助标准自然增长机制，救助困难群众42.4万人，惠及低保对象8.6万人。“泉房通”平台投入使用，基本建成保障性安居工程2.7万套，房地产业保持健康发展。规范职工公积金缴交比例，发放贷款57.5亿元。全市慈善机构筹集善款超5亿元。坚持教育优先战略。完成学前教育第二期三年行动计划和特殊教育三年提升计划，新增公办幼儿园学位6200个；创建义务教育管理标准化学校88所，建设青少年社会教育活动中心26个。全市51万名外来务工人员随迁子女与本地学生享受平等教育权利。推进“一校多区”“小片区管理”和农村薄弱学校“委托管理”等改革。制定职业院校专业建设“正负面清单”，入选应用型本科转型试点高校3所、示范性现代职业院校12所，福州大学石油化工学院建成投用。加快卫生计生事业改革发展。福医大附属二院东海院区等项目进展顺利，泉州滨海医院建成投用，全市新增医疗机构床位2000张。完成医保的管理经办“三保合一”，创新联合办医模式和“公建民营”办医体制。基层医疗卫生服务能力不断增强。实施“全面两孩”政策，推行基层计生工作规范化管理。推动文化传承发展。建成泉州木偶剧院，完善提升乡镇综合文化站27个，铺开文艺精品公益性演出，成为央视春晚、元宵晚会分会场。编制21个闽南文化重点区域保护规划，建立非遗生产性保护基地29个，建成工艺美术大师工作室130个。安溪获评中国藤铁工艺之都。成功承办第八届亚洲世界语大会。举办环泉州湾国际公路自行车赛。获得里约残奥会金牌一枚，实现夏季奥运会奖牌零的突破。持续推进生态文明建设。完成永春空间规划、晋江自然资源资产负债表编制等9项改革成果。141个重点流域、跨境流域污染整治项目完成投资14.9亿元，小流域“赛水质”活动整治河道69公里，所有集中式饮用水源地和主要干流水质达标率保持100%；空气优良率98.3%。禁养区内生猪养殖场基本关闭拆除。完成植树造林5.9万亩、水土流失治理40.6万亩、矿山整治恢复255万平方米。永春获评中国生态文明奖。深化平安建设。开展信访“五抓一促”活动，完善矛盾纠纷多元化解机制。强化社会治安综合治理，严厉打击各类刑事犯罪，集中治理电信网络诈骗，成为全国公共安全视频监控联网应用示范城市。推进诚信建设，建立激励和制约机制。认真执行市人大关于进一步加强食品安全监督管理议案的决议，较好完成各项治理目标任务，基本建立覆盖市县乡三级的食品药品监管体系，餐饮服务“明厨亮灶”工作经验在全省推广。深化安全生产标准化提升工程三年行动，开展对危化、交通、煤矿、消防、电梯、建筑施工等重点领域专项整治，事故指标均有下降。支持国防和军队建设改革，进一步规范国防动员准备运行机制，推动军民融合发展，拓展双拥工作，荣获全国双拥模范城“八连冠”和全国人民防空先进城市。民族宗教、统计、物价、方志、档案、气象、地震以及妇女儿童、青少年、残疾人、红十字会、工会等工作都取得新成效。

　　（六）转变作风加强政府自身建设

　　深入开展“两学一做”学习教育，推动政府作风建设常态化长效化。自觉接受市人大及其常委会监督，依法执行市人大及其常委会决定决议，主动配合市人大依法履职，办理人大代表建议357件，满意和基本满意率达100%。支持市政协履行职能，主动接受民主监督，深化重大事项事前协商，办理政协提案483件，满意和基本满意率达99.8%。坚持依法治市，落实府院联席机制，加强行政执法与刑事司法衔接，启动“七五”普法。深化“放管服”改革，进一步规范压缩项目推进全过程的自由裁量度，减少前置审批和中介服务事项124项，取消各类证照证明材料541件，清理规范行政审批申报得到国务院表扬推广。健全大督查机制，机关效能建设进一步深化。严控“三公”经费支出，完成全市党政机关公务用车制度改革任务。加强廉政建设、行政监察、审计监督等工作。

　　各位代表，过去五年，我们顶住了改革开放以来最严峻的经济下行压力，克服了转型升级的阵痛，成功战胜了自然灾害，成绩来之不易。这是省委、省政府和市委正确领导的结果，是全市人民齐心协力、团结奋斗的结果。在此，我谨代表市人民政府，向全市广大干部群众，人大代表、政协委员，各民主党派、工商联、无党派人士、各人民团体、离退休老同志，向驻泉省部属单位、驻泉部队、武警官兵，企业界人士和来泉创业务工人员，向广大港澳同胞、台湾同胞、海外侨胞、海内外朋友们，表示衷心的感谢和崇高的敬意！

　　同时，我们清醒地认识到，泉州发展中还存在不少困难和问题。主要是：产业转型处在“博弈过程、胶着状态”，传统产业艰难调整，高新技术产业、现代服务业规模偏小，新旧动能转换任重道远。企业“两极分化”加剧，部分企业生产经营困难，防范金融风险压力加大。城市品位内涵有待提升，内涝、交通拥堵、停车难等问题比较突出；农业农村基础较为薄弱。环境保护压力较大，应对重大自然灾害能力有待提升。优质教育、医疗、养老资源供给相对不足，多元公共服务与人民群众新期待还有不少差距。政府职能转变不够到位，营商环境有待进一步改善。对此，我们要高度重视，继续努力加以解决。

　　二、新一届政府工作及2017年工作目标任务

　　今后五年，是全面建成小康社会的决胜期，也是泉州加快发展的难得机遇期。政府工作指导思想是：深入贯彻习近平总书记系列重要讲话精神和对福建工作的重要指示，全面贯彻党的十八大和十八届三中、四中、五中、六中全会精神，牢固树立“四个意识”特别是核心意识和看齐意识，紧紧围绕“五位一体”总体布局和“四个全面”战略布局，认真落实省市党代会部署，积极践行新发展理念，勇当建设“新福建”领头羊，全力推进创新驱动发展、供给侧结构性改革，全力推进经济扩量提质、城市内涵集聚、民生事业提升，加快建设“创新、智造、海丝、美丽、幸福”的现代化泉州。

　　今后五年的总体目标和重要任务是：在实现“十三五”规划目标的基础上，一是迈向万亿经济强市行列。加快产业结构战略性调整，致力打造国家自主创新示范区、“中国制造2025”城市试点示范。坚持路线图引领，巩固提升纺织鞋服、石油化工、机械装备、建材家居4个主导产业，加快发展食品、工艺制品、纸业印刷3个特色产业，培育智能装备、新一代信息技术和半导体、交通运输装备、金融服务、大旅游、大物流、大健康7个500亿～1000亿元的新集群。力争经过五年努力，制造业基本形成传统优势产业、高新技术产业、重化产业三大板块格局，第三产业增加值占GDP比重达40%。二是建设“海丝”重要门户城市。实施千年古港复兴计划，进一步活化“海丝”基因，保护“海丝”文化遗产，做好牵头“海丝”申遗工作。坚持古城提质，实施整体性保护、微扰动改造、低冲击更新、家园式管理，让古城“见人、见物、见生活”。坚持新城集聚，推动环湾规划建设、基础设施、产业布局和城市功能协调衔接，加快建设“海丝”新城。按照省域三大中心城市定位，下决心推动行政区划调整，统筹“一湾、两江、两翼”空间发展。三是共建共享美丽幸福泉州。致力补齐民生短板，推动教育、医疗、养老、社保、文化等基本公共服务供给普惠共享。落实扶贫攻坚“六个精准”，确保贫困人口全部脱贫、贫困村全部“摘帽”。完善生态文明制度体系，大力发展绿色、循环、低碳经济，打好水、大气、土壤污染防治攻坚战。全力提升社会治理现代化水平，切实维护社会和谐安定。四是打造一流营商环境。发挥国家级“金改区”“综改区”“海丝”先行区、自主创新示范区、生态文明试验区等“五区叠加”的改革效应，再创体制机制新优势；不断赋予“泉州模式”“晋江经验”新内涵，构建“亲”“清”新型政商关系，努力把泉州建设成行政审批事项最少、行政效能最高、企业运营成本最低的城市之一。

　　2017年是新一届政府的第一年，是“稳中求进、突围攻坚、注重长远”的关键一年。“稳中求进”是总基调，要大力振兴实体经济，通过政企联手、巩固工业、大抓三产，稳信心、稳速度、稳大盘，力求创新有进、结构有优、质量有升。“突围攻坚”是军令状，要着力在产业转型升级、重大项目投建、金融风险防范、城市集聚提质、民生事业补短等方面打攻坚战、啃硬骨头，拿出成果、拉开格局。“注重长远”是着眼点，以功成不必在我的责任担当，接续奋斗，接力发展，多做打基础的工作，多抓增后劲的项目，多办利长远的实事。2017年主要预期目标是：全市生产总值增长8%，农业总产值增长2.5%，工业增加值增长7.6%，第三产业增加值增长9%；一般公共预算总收入增长5%，一般公共预算收入增长5%；固定资产投资增长13%；实际利用外资增长2%，出口增长2%；社会消费品零售总额增长11%，居民消费价格涨幅控制在3%左右；居民人均可支配收入增长7%；完成节能减排任务。重点抓好以下七方面工作：

　　（一）更加注重创新驱动，积极构筑现代产业版图

　　实体经济是泉州的根基。坚持做大总量与提升质量并重，推进制造业与服务业双轮驱动、融合并进，让实体经济始终扎根泉州、更好发展。

　　深入实施产业转型升级路线图。坚持“一图一策”，全力推动产业向中高端迈进。始终把传统优势产业作为百年基业。突出用新技术新业态改造提升传统产业，在持续推进“机器换工”的同时，跟进新技术扩散应用，率先进入石墨烯、可穿戴设备、智能家居等前沿领域；突出优化产业组织，发挥龙头企业引领带动作用，引导中小企业专注代工、定制、配套等，抓好泛家居联盟、品牌工业园、建筑产业现代化基地等项目；突出营造产业生态，强化供应链管理、融资保障、公共技术平台等支撑，巩固提升传统优势产业在国内外的话语权。着力壮大重化产业。抓好中化乙烯、天佑能源、天骄化学材料等重大项目，加快向精细化工等下游产业链延伸，建好泉港、泉惠石化园区。大力发展高新技术产业。在集成电路、光电光伏、高新材料、微波通讯、工业机器人等领域，打造一批具有较强创新实力的产业集群。加快规划建设泉州“芯谷”，以晋华集成电路、三安系列项目为龙头，用心培育设计、制造、封测、终端应用等全产业链，建好晋江、安溪、南安三大园区。加快现代服务业提质扩量。开展“第三产业提升年”活动，致力把制造业大市、深厚文化底蕴优势，转化为服务业发展的现实生产力。一是突出“生产基地+物流+互联网”。立足庞大制造业货源优势，鼓励龙头企业建设智能仓配一体化园区；抓好中农批国际物流港、泉港石化物流贸易基地、菜鸟智能物流园区等项目，培育第三方、第四方物流；扶持建设优势产业垂直供应链电商平台，运营好“泉州购”“跨境通”。加快发展与制造业相配套的工业设计、金融服务、会展等生产性服务业。二是突出“海丝+古城+旅游”。主打“清新福建·海丝泉州”旅游品牌，以“海丝”申遗为契机，以“一城、一山、一港、一馆、一镇”为抓手，策划实施一批“海丝”旅游项目、路线、产品，全域化发展古城旅游；培育美丽乡村旅游、滨海休闲度假旅游和工业、商务、宗教、探亲旅游，抓好台商区大型海洋生态、南安冰雪大世界、惠安闽台影视基地、安溪新奥北部湾、永春乡愁公园、德化石牛山等旅游项目。实施服务业“品质革命”，推动健康养老、文体娱乐、休闲养生、家政护理等向精细和高品质转变。

　　加快国家自主创新示范区建设。编制实施泉州片区发展规划纲要，致力在创新和示范上下功夫，让各种创新要素在这里落地生根、开花结果，让泉州成为最具吸引力的创业创新乐园。一是突出体制机制创新。完善示范区配套政策，实施重点项目20个以上，不断创新科研经费使用、科研评价考核、科技金融结合、科技成果转化、知识产权运用等体制机制，努力在引导民间资本投入科技创新上树立典范。二是突出产业创新转型。发展壮大新兴产业，引导传统产业开发应用新技术、新工艺、新材料、新产品，培育一批高新技术企业、科技小巨人领军企业。开展高新区争先进位行动，力争高新区工业产值增长15%以上。三是突出汇聚创新资源。继续采取“一院一策”“实体办院、投管分离、专业化运营” 等方式，引进国内外院校来泉设立科技创新平台，支持与本地企业开展产学研合作。实施“海纳百川”高端人才集聚计划和引进人才精准政策包。深化企业家素质提升培训计划，重点支持新生代传承创新，培育众创空间、孵化器10家以上。

　　开展“中国制造2025”城市试点示范。制定总体实施方案和支撑政策体系，主动承接国家重大工程和试点项目，努力建设先进制造业强市。一是加快推进智能化数控化改造。实施智能制造示范创建工程，抓好九牧智能制造、恒安智能化生产（二期）等项目，新建智能化车间18个，培育示范产品70个以上；实施重点技改项目200个以上，规上企业数控化率提升至45%。二是打造全国重要的智能轻工装备基地。立足纺织、鞋服、食品、建材等产业升级需求，引导发展与之相配套的智能装备和工业机器人，抓好嘉泰数控（二期）、通达智能制造（二期）、正丰数控等项目，建设洛江、晋江、南安智能装备产业园。大力发展服务型制造，促进装备制造产业与云计算、物联网、移动互联网相结合，推动企业由单一产品制造，向成套设备集成、生产线设计、供应链服务等提供商转型，建设制造云平台。三是树好“泉州制造”品牌形象。大力创建全国质量强市示范城市，实施质量品牌提升计划，开展“泉州制造”品牌认证，推进泉州商标馆建设。引导企业发扬“工匠精神”，争创国际品牌，打造“百年老店”。举办第三届“海丝”国际品牌博览会。

　　全力抓项目促投资。组织开展“项目攻坚年”活动，加快“五个一批”和四个领域补短板项目库提质扩容，建立挂图作战、问题销号制度，推行代建制，确保项目快速推进。实施在建重点项目396个、总投资8245亿元，年度计划投资1158亿元，开工重大项目70个、竣工70个。充分发挥本地民间资本的积极性，坚持“引资”与“引智”“引技”相结合，拓展“政民外”对接，强化招商考核，全力引进一批产业龙头项目。

　　（二）更加注重强农惠农，大力发展特色现代农业

　　深化农业供给侧结构性改革。一是推进标准生产。大力发展设施农业，建设种植业标准化示范片147个，培育果蔬大棚2000亩、生态茶园1.2万亩，实施 “一区两园”项目20个，支持中科生物、道畋等龙头企业发展。二是推进品牌建设。实施农产品质量安全“1213”行动计划，新增“三品一标”认证20个以上，打造更多名牌农产品，让人民群众吃得安全、吃得放心。三是推进专业经营。新增农民合作社300家，扶持示范社30家，认定新型职业农民1000名；有序推进农村土地经营权流转，基本完成农村承包土地确权登记颁证工作，抓好农村“两权”抵押贷款试点。四是推进融合发展。突出“接二连三”，扶持30家生产加工和流通型龙头企业，加强冷链物流建设，扶持发展林下经济、渔港经济、休闲农业和田园风光。壮大农产品电商平台，培育10家农业物联网示范企业。创新粮食储备与加工相结合的机制，抓好市区中心粮库等11个项目。

　　建设美丽乡村。实施新一轮“千村整治、百村示范”，突出农村卫生保洁和污水治理，抓好一批环境整治村、示范宜居村和美丽乡村景观带建设。实施水利项目150个，开工白濑水利枢纽工程，推进七库连通、马跳水库、南安“两溪一湾”整治等重大项目建设，抓好农村饮水、海堤加固、水库除险等民生水利。持续推进农村电网升级，实施公路防护工程300公里、危病桥改造15座以上。

　　打好脱贫攻坚战。市财政统筹1.15亿元，扶持30个市级扶贫重点乡镇、300个扶贫重点村发展。综合运用部门挂钩、干部驻村、捆绑资金、项目带动等方式，帮扶一批重点村脱贫发展和村集体经济创收，努力解决大病致贫、大病返贫问题。实施造福工程搬迁4000人，实现农村贫困人口全部脱贫。

　　（三）更加注重试点牵引，纵深推进改革开放攻坚

　　抓好重大改革试验。紧紧抓住全国新一轮改革开放的重大机遇，进一步放大“金改”“综改”等国家级改革试点的示范效应。始终坚持金融服务实体经济的本质要求，加大金融产品和服务创新，完善提升泉州金融服务平台、中小微企业信用信息交换共享平台，拓展全产业链金融服务。深化对台对外金融合作。抓好泉州（东海）金融供应链集中区、晋江金融广场、泉州高新区科技金融服务中心等项目，做大做强泉州银行，加快民营银行申报。争取获批移动互联网支付牌照。坚持化风险和促发展并举，攻克重点行业、重点区域风险点，依法从严从重打击恶意逃废债，维护良好金融生态。发挥海峡股权泉州交易中心的综合投融资服务功能，支持一批优质企业改制上市，加快兼并重组、城市发展、不良资产处置等基金落地运作，大力发展融资租赁业务，拓宽民间资本进入金融领域、实体经济通道。深化工商登记制度改革，继续推进国家级电子证照共享平台建设。全面放开竞争性领域和环节的价格，政府定价范围主要限定在重要公用事业、公益性服务、网络型自然垄断环节。按照“资产集中、经营细分”原则，加强国资布局规划，抓好金控、城建、文旅集团运营，做大做强国有企业。推进供销社、林业等改革。

　　拓展对外开放的广度和深度。深入实施“海丝”先行区建设行动方案和发展规划。更加注重外贸发展质量和内涵，坚持走出去营销和引进来采购相结合，发挥专业会展、专业市场、电商平台作用，大力培育外贸综合服务型企业，支持自主品牌和高附加值产品出口。有序引导合适产能走出去，推动民营企业组团赴境外设立生产基地、商品交易中心，带动设备、材料输出，引导资源、资金回流，培育本土跨国公司。推动泉州开发区新老园区连片发展，发挥综合保税区、陆地港的政策功能优势，加强与海港、空港的资源整合、互动发展。抓好石湖5～6#泊位、石井16～17#泊位、泉州晋江国际机场改扩建等项目，大力推进港后区规划建设，推行物流全程“一单制”，增开内外贸航班航线，争取更多货源从泉州港进出。

　　深化与台港澳侨交流合作。高标准开发建设台商区，力争在引进重大产业项目、高端创新平台上取得突破。深化对台民间交流交往，推动客货运直航发展壮大，实现向金门供水工程正式通水。密切与港澳侨经贸文化交流，加快南洋华裔族群寻根谒祖平台建设，涵养新华侨华人和华裔新生代资源。

　　（四）更加注重全域集聚，做大做强环湾中心城市

　　古城要做好“留”的文章。坚持全城保护、修旧如旧，开展城市修补、生态修复，做到“留形、留神、留人”。集中力量推进城南、府文庙、龙头山、华侨新村四个片区的规划设计、修缮整治和业态活化。抓好中山中路步行化改造、泉州美食城、东亚文化创意产业园、八卦沟景观提升等项目。举办第二届“海丝”古城徒步穿越活动。推广社区营造示范试点，建立家园共造机制。

　　新城要做好“聚”的文章。强化统筹意识，做好拼图作业，全力打造“东海会展中心—滨海公园（公共文化中心）—后渚片区—城东”的沿江滨海城市展示面，集中力量建设“海丝”新城。一是实施综合交通提升行动。加快打通环湾“大动脉”，推进泉厦漳城市联盟路、城东至北峰快速通道、综合交通枢纽等项目建设，开工兴泉铁路、福厦客专、有轨电车，建成厦沙高速德化段、安溪段。发展城市交通微循环，推进重要节点渠化改造。完善城际公交、社区公交、公共自行车和步行系统，推行公交一元票价制，加强公共停车场建设。二是实施生态景观建设提升行动。强化市区城中山保护管理，基本建成山线示范段、滨江北路水线，实现泉州植物园部分开放。深化晋江下游生态整治，提升“两江”沿岸、城市门户廊道、主干道景观，建设一批“口袋公园”、袖珍公园。三是实施公共配套设施提升行动。进一步完善供水、供气、排水系统，开工建设金鸡水厂；推进内涝防治工程，抓好海绵城市试点；加强污水收集处理，做好中心市区内沟河截污，逐步建立雨污分流体系；大力推进垃圾分类处理利用。同步配套教育医疗、公共文体、社会保障等设施。

　　管理要做好“精”的文章。全面开展城市设计，制定环湾规划负面清单，落实规划审批报备制度，完成“多规合一”试点。实施城市管理提升行动，整合市容环卫、市政公用、园林绿化、综合执法等职能，构建“大城管”格局。全面推行精细化管理，强化中心市区文明施工，规范道路开挖修复，建设西街、中山路等管理示范街，开展农贸市场标准化改造，确保通过全国文明城市、国家卫生城市复审。加快“智慧城市”建设，突出数据交换共享，推进“数字城管”向“智慧城管”升级。进一步提升社区治理和物业管理水平。

　　新型城镇化要做好“人”的文章。围绕区域协同、城乡一体，科学布局“一湾两翼”的生产、生活、生态空间。突出产城融合，坚持产业聚人、功能留人，高标准规划建设南北翼新城，抓好石井半导体园区、泉港石化园区安全控制区等项目；推进县域大城关扩容提质，统筹引导山区生态经济连片发展。深化试点小城镇建设，制定特色村镇培育政策，加快创建一批“产、城、人、文”四位一体有机结合的特色小镇。坚持“房子是用来住的、不是用来炒的”定位，加快建立房地产平稳健康发展长效机制。支持农业转移人口市民化，提高户籍人口城镇化率。

　　（五）更加注重文化引领，着力增强城市发展软实力

　　传承千年的多元文化、闽南文化，是泉州人最美的乡愁。要用“活化”保护的方式，让千年文化更生动地活在古街古巷中，活在市井生活中，活在旅游经济中。

　　全力以赴申报“海丝”世界文化遗产。发挥牵头城市的引领作用，抓好《泉州市海上丝绸之路史迹保护条例》的宣传和实施，高标准、高质量完成“海丝”遗产点保护修缮、环境整治、展示利用等各项申报任务。启动中国海上丝绸之路博物馆建设。办好第三届“海丝”国际艺术节。

　　实施历史文化名城保护行动。注重延续名城整体风貌格局，加强对历史文化街区、名镇、名村及传统村落的保护提升。深入推进闽南文化生态保护重点区域建设，抢救保护濒危非遗代表性项目，改造提升泉州非遗馆，推进非遗数字化，争创一批国家级、省级非遗生产性示范基地。支持文艺精品创作。系统研究和挖掘历史名人文化。实施泉州老字号振兴计划。落实文物平安工程，做好东关桥等受损文物修复。

　　完善公共文化服务。持续开展文化惠民“万千百十”工程，抓好市公共文化中心、泉州歌舞剧院等项目建设。完善提升县镇村三级公共文化设施，打造24个基层综合性文化服务中心示范点。实施全民健身计划，加强老年体育工作。支持体育用品企业抢占细分市场，培育发展体育服务业，进一步提升环泉州湾国际公路自行车赛的层次和影响力。

　　（六）更加注重绿色低碳，加快建设生态文明试验区

　　绿色是大自然的底色，是人民群众的期盼。要始终把生态文明理念深植于施政全过程，为子孙后代留住蓝天净土、绿水青山。

　　创新生态文明体制机制。大力推进国家生态文明试验区建设，确保完成环境污染强制责任保险、项目环境影响评价信息公开等12项综合试验任务，努力形成一批在全省、全国可复制推广的成果和经验。落实环保“党政同责、一岗双责”，进一步加强重点生态功能区、生态红线管控区保护，严格生态环境损害责任终身追究制，抓好国家环保督察迎检，组织开展市对县环保督察。

　　深化环境污染综合整治。全面落实水、大气、土壤污染防治行动。加强全市流域水环境保护，实施重点流域、跨境流域、小流域“赛水质”整治项目100个，年度计划投资10亿元；深入推进“河长制”管理，促进河道管护常态化、制度化，完善下游对上游的生态补偿机制，建设安全生态水系。持续推进禽畜养殖场达标改造。加强空气质量预警预报，狠抓工业粉尘、餐饮油烟、建筑施工等污染源治理，完成黄标车淘汰任务。加快工业废物综合处置中心、医疗废物应急处置中心投用，实现危险废物快速、安全处置。加强水土保持、造林绿化，抓好矿山、海岸线修复，争创国家森林城市。

　　突出资源节约集约利用。严格落实项目能评制度，完善重点用能企业的能源监测、审计和管理体系，开展节能量交易试点。加快淘汰落后产能，完成燃煤锅炉节能环保提升任务，关停年产9万吨以下的小煤矿，推进德化陶瓷、泉港石化、石狮染整等循环经济园区建设。坚守耕地保护红线，依法处置闲置土地和盘活城乡低效建设用地。争创国家节水型城市。

　　（七）更加注重补齐短板，不断强化民生托底保障

　　解民忧、惠民生的事情慢不得、省不得。要把民生投入作为首要保障，扎实抓好养老、教育、卫生三大民生建设，不断刷新惠民高度和群众幸福指数。

　　大力发展以居家为基础的养老服务。出台实施城乡社区居家养老服务的政策措施，推动养老服务改革创新，发展壮大养老服务业。积极引进社区居家养老专业化服务组织，构建养老服务信息化平台。突出医养结合，抓好养老服务工程包，推动江南老年颐乐园提升运营，建设市社会福利中心、晋江社会福利中心（二期），引导社会力量兴建养老机构。

　　办好人民满意的教育。全面普及学前三年教育、高中阶段教育，新扩建公办幼儿园30所，抓好中小学建设工程包，创建义务教育管理标准化学校250所。实施名校长、名教师、名班主任培养工程。深化职业院校专业群与产业群对接，培育一批特色专业群，设立一批职业院校联盟、职教集团、校企深度合作公共实训基地，创建示范性现代职业院校，抓好应用型本科转型试点高校建设。壮大“双师型”教师队伍，推广现代学徒制，大力培养高技能人才。继续抓好老年教育，建设老年大学新校区。发展特殊教育、终身教育。扶持和规范民办教育。持续加强和改进未成年人思想道德建设，做好关心下一代工作。

　　加快卫生计生事业发展。全力推进优质医疗资源扩容，扩建续建福医大附属二院东海院区、第一医院城东新院、医高专附属人民医院、光前医院、儿童医院，新建老年医院、妇产医院、颐和医院，支持社会办医，新增医院床位2500张以上。改革医保支付方式，实现城乡居民基本医保一体化。深化公立医院综合改革，建立完善院长目标年薪制和医务人员薪酬制度，大力引进培养卫生人才。推行分级诊疗模式，加强县级公立医院五大核心专科和五大薄弱学科建设，开展基层综合医改，推广乡村卫生服务一体化管理和城市家庭医生签约服务。加强重大疾病和慢性病防控。创建全国中医药先进市。落实计生目标管理责任制，完善“全面两孩”配套政策，提升妇幼健康服务能力；推动计生服务管理转型发展，充分发挥计生协会作用。

　　提高社会保障水平。实施就业促进和创业引领计划，推动高校毕业生、退伍军人和城乡就业困难人员，实现更高质量就业创业。推进全民参保登记，稳步提高各项社会保险待遇水平。抓好保障性安居工程，加快石结构房屋、农村危房改造，推广征迁安置货币化补偿。推行低保标准、扶贫标准“两线合一”，深化泉港、晋江“救急难”试点。重视发展社会救助、慈善事业。深入实施妇女儿童发展纲要，完善留守儿童保障体系。

　　加强和创新社会治理。认真落实信访工作责任制，依法、及时、就地解决信访问题，引导群众采用行政复议、行政诉讼渠道解决行政争议。全面深化公安改革，加强社会治安综合治理，依法严厉打击违法犯罪活动，完善治安防控立体化体系。强化守信激励和失信惩戒联动机制，建设诚信社会。落实食品药品安全地方政府负总责，按照“四个最严”要求，加大食品药品安全监管执法力度，实施重点区域、重点品种食品安全风险监测，创建国家食品安全城市。抓好道路交通、消防、危化、建筑施工等重点领域安全生产，深化隐患排查治理体系建设，创建国家安全发展示范城市。支持工会、共青团、妇联等人民团体发挥更大作用。深入开展民族团结进步创建活动。加强宗教事务依法管理。对接落实国防和军队改革任务，深入推进军民融合发展，抓好随军家属就业、子女入学等工作，不断完善双拥运行机制，巩固发展军政军民团结良好局面。

　　三、全面提升政府的施政能力和服务水平

　　勇挑重担、精心运作。拿出踏石留印、抓铁有痕的韧劲和恒心，提振精气神，勇当领头羊，推动泉州发展行稳致远。坚持路线图思维，每项重要工作都要整体谋划、找准路径、近中远推进。坚持项目化管理，以抓项目的方法推进政府工作，定目标、定进度、定考核，一抓到底，抓出成效。坚持专业化推进，在工作中锤炼一支懂业务、想干事、干成事的干部队伍。健全激励机制、容错机制，鼓励敢闯敢试、首创独创，形成比学赶超的浓厚氛围。

　　厉行法治、依法行政。坚持用法治思维、法治方式履行职责，推动政府工作全面纳入法治轨道。做好政府立法工作，推进“七五”普法，拓展府院良性互动内涵。自觉接受人大的法律监督、工作监督和政协的民主监督，提高人大代表建议和政协提案的办理质量，充分听取各民主党派、工商联、无党派人士以及各人民团体的意见和建议，广泛接受新闻舆论和人民群众监督。

　　简政放权、服务于民。经济下行压力越大，越要主动靠前服务；财政增收压力再大，也要优先保障民生。把规范压缩自由裁量权，作为深化“放管服”改革的突破口，持之以恒抓出特色，打造成为政府服务的亮丽品牌。进一步发挥政企互动的好传统，保护企业家精神，不折不扣落实惠企政策，坚决整治侵害企业利益的不正之风。高标准实施33件为民办实事项目，努力把市民的期待变成我们的行动，把市民的希望变成生活的现实。

　　正风肃纪、廉洁自律。按照全面从严治党要求，把纪律和规矩挺在前面，认真落实党风廉洁建设责任制，严格执行党内政治生活若干准则、廉洁自律准则和纪律处分条例、问责条例、党内监督条例，严守中央八项规定。完善公共资源市场化配置、全口径预算管理、政府购买服务、政务公开、国企监管等制度，有效消除权力寻租空间。加强行政监察和审计监督，深化效能建设。

　　各位代表，新的征程已经开启，宏伟蓝图催人奋进。让我们紧密团结在以习近平同志为核心的党中央周围，在中共泉州市委领导下，与全市人民一道，不忘初心、继续前进，奋力开创“五个泉州”新局面，以优异成绩迎接党的十九大胜利召开。

　　各位代表，泉州人有精神，企业有根，社会有活力。让我们按照习近平总书记的要求，撸起袖子加油干吧！
